# Supplementary material for: Odor mixtures of opposing valence unveil inter-glomerular crosstalk in the Drosophila antennal lobe
Source: Nat Commun. 2019 Mar 13;10:1201. doi: 10.1038/s41467-019-09069-1 (PMC6416470; doi:10.1038/s41467-019-09069-1)
Supplement: Supplementary file 1 — Supplementary Information [file 41467_2019_9069_MOESM1_ESM.pdf]

Supplementary Information

Odor mixtures of opposing valence unveil inter-glomerular crosstalk in the *Drosophila* antennal lobe

Mohamed et al.

Supplementary Table 1. Transgenic flies used in this study.

Figures	Genotypes
Fig. 1	Wild-type Canton S
Fig. 2 (except for 2g,i)	*w-; GH146-Gal4,UAS-GCaMP6s/(Cyo); TM2/TM6B*
Fig. 2g,i	Wild-type Canton S
Fig. 3a-d	Wild-type Canton S
Fig. 3a',a'', 3b',b'', 3c'-3c''',3d'-3d'''	*w-; GH146-Gal4,UAS-GCaMP6s/(Cyo); TM2/TM6B*
Fig. 4b,c	*$Or10a^{f03694}$; GH146-Gal4,UAS-GCaMP6s/(Cyo); TM2/TM6B*
Fig. 4e,f	*$Or7a^{-/-}$; GH146-Gal4,UAS-GCaMP6s/(Cyo); TM2/TM6B*
Fig. 4h	*Canton S, w^{1118}, $Or10a^{f03694}$; +/+; +/+ and $Or7a^{-/-}$; +/+; +/+*
Fig. 5b,c	*w-; Or10a-Gal4; GH146-QF,QUAS-GCaMP3/20x UAS-CsChrimson-mCherry-trafficked (VK00005)*
Fig. 5e,f	*w-; 20x UAS-CsChrimson-mCherry-trafficked (in su(Hw)attP5); GH146-QF,QUAS-GCaMP3/Or7a-Gal4*
Fig. 5g	as mentioned in the figure
Fig. 6	*w-; GH146-Gal4,UAS-GCaMP6s/(Cyo); TM2/TM6B*
Fig. 7b,c	*UAS-dicer2; GH146-Gal4,UAS-GCaMP6s/UAS-empty RNAi; TM2/TM6B*<br>*UAS-dicer2; GH146-Gal4,UAS-GCaMP6s/UAS-GBi; UAS-GBi/TM6B*<br>*UAS-dicer2; GH146-Gal4,UAS-GCaMP6s/(Cyo); UAS-Rdli/TM6B*<br>*UAS-dicer2; GH146-Gal4,UAS-GCaMP6s/UAS-gluclalpha RNAi; TM2/TM6B*
Fig. 7e,f	*UAS-dicer2; UAS-empty RNAi/Cyo; GH146-QF,QUAS-GCaMP3/Orco-Gal4*<br>*UAS-dicer2; UAS-GBi /(Cyo); GH146-QF,QUAS-GCaMP3/Orco-Gal4*<br>*UAS-dicer2; Orco-Gal4/(Cyo); GH146-QF,QUAS-GCaMP3/UAS-Rdli*<br>*UAS-dicer2; UAS-gluclalpha RNAi/(Cyo); GH146-QF,QUAS-GCaMP3/Orco-Gal4*
Fig. 8a-d	*UAS-dicer2; UAS-empty RNAi/(Cyo); UAS-GCaMP6f/ GH298-Gal4*<br>*UAS-dicer2; UAS-Gad RNAi/(Cyo); UAS-GCaMP6f/ GH298-Gal4*<br>*UAS-dicer2; UAS-empty RNAi/(Cyo); UAS-GCaMP6f/ NP3056-Gal4*<br>*UAS-dicer2; UAS-Gad RNAi/(Cyo); UAS-GCaMP6f/ NP3056-Gal4*<br>*UAS-dicer2; UAS-empty RNAi/(Cyo); UAS-GCaMP6f/ H24-Gal4*<br>*UAS-dicer2; UAS-Gad RNAi/(Cyo); UAS-GCaMP6f/ H24-Gal4*<br>*UAS-dicer2; UAS-empty RNAi/(Cyo); UAS-GCaMP6f/ HB4-93-Gal4*<br>*UAS-dicer2; UAS-Gad RNAi/(Cyo); UAS-GCaMP6f/ HB4-93-Gal4*
Fig. 8a'-d'	*UAS-dicer2; UAS-empty RNAi/(Cyo); GH146-QF,QUAS-GCaMP3/GH298-Gal4*<br>*UAS-dicer2; UAS-Gad1 RNAi/(Cyo); GH146-QF,QUAS-GCaMP3/GH298-Gal4*<br>*UAS-dicer2; UAS-empty RNAi/(Cyo); GH146-QF,QUAS-GCaMP3/NP3056-Gal4*<br>*UAS-dicer2; UAS-Gad1 RNAi/(Cyo); GH146-QF,QUAS-GCaMP3/NP3056-Gal4*<br>*UAS-dicer2; UAS-empty RNAi/(Cyo); GH146-QF,QUAS-GCaMP3/H24-Gal4*<br>*UAS-dicer2; UAS-Gad1 RNAi/(Cyo); GH146-QF,QUAS-GCaMP3/H24-Gal4*<br>*UAS-dicer2; UAS-empty RNAi /(Cyo); GH146-QF,QUAS-GCaMP3/HB4-93-Gal4*<br>*UAS-dicer2; UAS-Gad1 RNAi/(Cyo); GH146-QF,QUAS-GCaMP3/HB4-93-Gal4*
Fig. 8e,f	*w-; UAS-C3PA-GFP/+ ; HB4-93/Gal4/GH146-QF, QUAS-mtdTomato*<br>*w-; UAS-C3PA-GFP/(Cyo) ; NP3056-Gal4/GH146-QF, QUAS-mtdTomato*
Fig. 8g,h	*w-; UAS-homer-GCaMP3/+; NP3056-Gal4/+*<br>*w-; UAS-homer-GCaMP3/+; HB4-93-Gal4/+*<br>*UAS-Syt::HA; UAS-mCD8-GFP/(Cyo); NP3056-Gal4/+*<br>*UAS-Syt::HA; UAS-mCD8-GFP/(Cyo); HB4-93-Gal4/+*

Supplementary Figures	Genotypes
Suppl. Fig. 2,3	*w-; GH146-Gal4,UAS-GCaMP6s/(Cyo); TM2/TM6B*
Suppl. Fig. 4a	*Or10a^{f03694}; GH146-Gal4,UAS-GCaMP6s/(Cyo); TM2/TM6B*
Suppl. Fig. 4b	*Or7a$^{-/-}$; GH146-Gal4,UAS-GCaMP6s/(Cyo); TM2/TM6B*
Suppl. Fig. 4c	as mentioned in the figure
Suppl. Fig. 5a-e	*w-; Or10a-Gal4; GH146-QF,QUAS-GCaMP3/20x UAS-CsChrimson-mCherry-trafficked (VK00005)*
Suppl. Fig. 5f-j	*w-; 20x UAS-CsChrimson-mCherry-trafficked (in su(Hw)attP5); GH146-QF,QUAS-GCaMP3/Or7a-Gal4*
Suppl. Fig. 6	*w-; GH146-Gal4,UAS-GCaMP6s/(Cyo); TM2/TM6B*
Suppl. Fig. 7a	as mentioned in the figure
Suppl. Fig. 7b,c	*UAS-dicer2; GH146-Gal4,UAS-GCaMP6s/UAS-empty RNAi; TM2/TM6B*<br>*UAS-dicer2; GH146-Gal4,UAS-GCaMP6s/UAS-GBi; UAS-GBi/TM6B*<br>*UAS-dicer2; GH146-Gal4,UAS-GCaMP6s/(Cyo); UAS-Rdli/TM6B*<br>*UAS-dicer2; GH146-Gal4,UAS-GCaMP6s/UAS-gluclalpha RNAi; TM2/TM6B*
Suppl. Fig. 7d,e	*UAS-dicer2; UAS-empty RNAi/Cyo; GH146-QF,QUAS-GCaMP3/Orco-Gal4*<br>*UAS-dicer2; UAS-GBi /(Cyo); GH146-QF,QUAS-GCaMP3/Orco-Gal4*<br>*UAS-dicer2; Orco-Gal4/(Cyo); GH146-QF,QUAS-GCaMP3/UAS-Rdli*<br>*UAS-dicer2; UAS-gluclalpha RNAi/(Cyo); GH146-QF,QUAS-GCaMP3/Orco-Gal4*
Suppl. Fig. 8a,b	*w-; Cyo/Bl; Orco-Gal4/UAS-GCaMP6f*
Suppl. Fig. 8c,d	*Wild-type Canton S*
Suppl. Fig. 9a	*UAS-dicer2; UAS-empty RNAi/(Cyo); GH146-QF,QUAS-GCaMP3/GH298-Gal4*<br>*UAS-dicer2; UAS-Gad1 RNAi/(Cyo); GH146-QF,QUAS-GCaMP3/GH298-Gal4*<br>*UAS-dicer2; UAS-empty RNAi/(Cyo); GH146-QF,QUAS-GCaMP3/NP3056-Gal4*<br>*UAS-dicer2; UAS-Gad1 RNAi/(Cyo); GH146-QF,QUAS-GCaMP3/NP3056-Gal4*<br>*UAS-dicer2; UAS-empty RNAi/(Cyo); GH146-QF,QUAS-GCaMP3/H24-Gal4*<br>*UAS-dicer2; UAS-Gad1 RNAi/(Cyo); GH146-QF,QUAS-GCaMP3/H24-Gal4*<br>*UAS-dicer2; UAS-empty RNAi /(Cyo); GH146-QF,QUAS-GCaMP3/HB4-93-Gal4*<br>*UAS-dicer2; UAS-Gad1 RNAi/(Cyo); GH146-QF,QUAS-GCaMP3/HB4-93-Gal4*
Suppl. Fig. 9b,c	*w-; UAS-homer-GCaMP3/+; NP3056-Gal4/+*<br>*w-; UAS-homer-GCaMP3/+; HB4-93-Gal4/+*<br>*UAS-Syt::HA; UAS-mCD8-GFP/(Cyo); NP3056-Gal4/+*<br>*UAS-Syt::HA; UAS-mCD8-GFP/(Cyo); HB4-93-Gal4/+*

SUPPLEMENTARY FIGURES 1-9

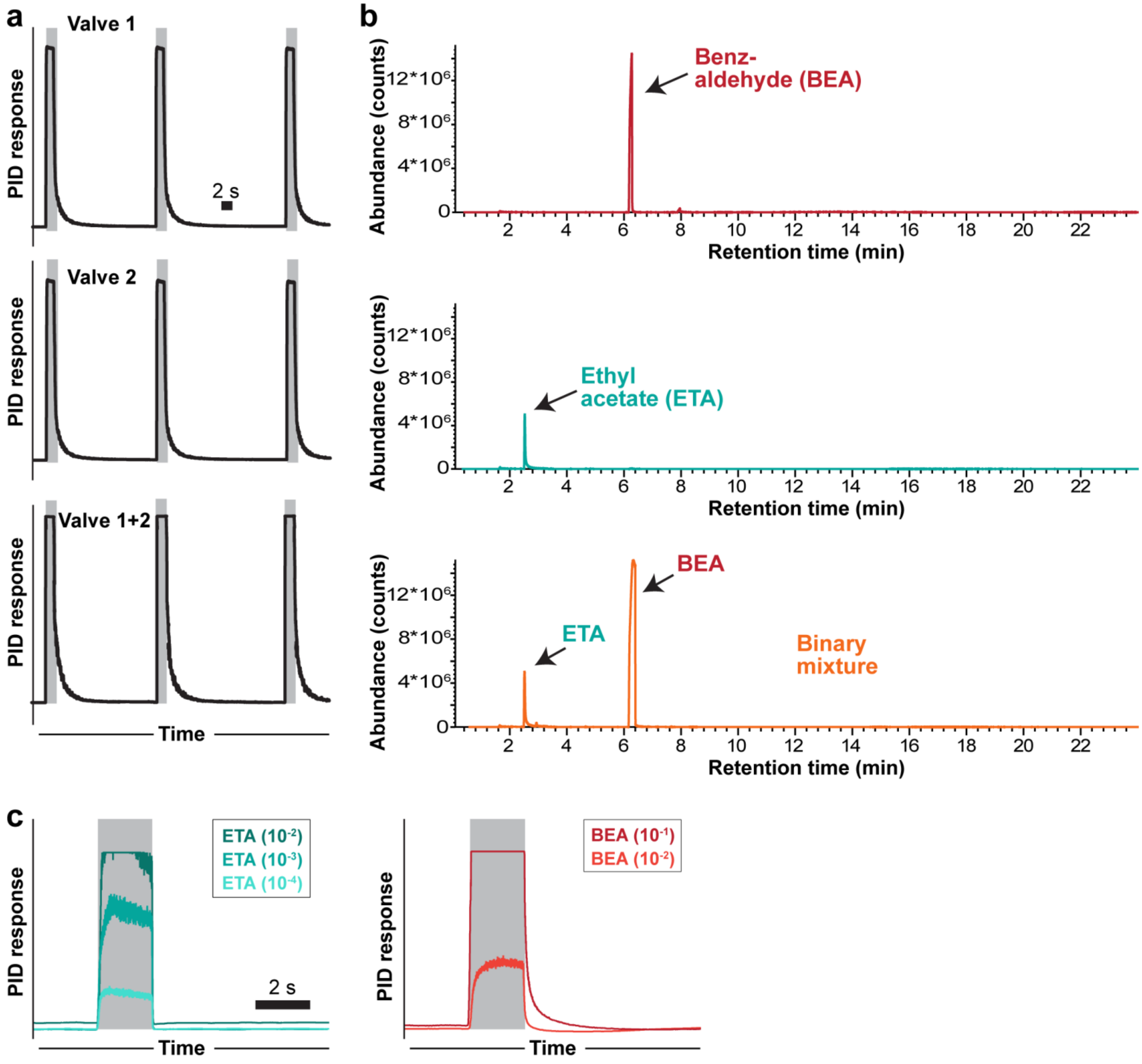

Supplementary Figure 1

Verification of odor delivery with PID and SPME GC-MS.

(**a**) Representative odor signals measured at the outlet of the odor delivery system (shown in Figure 2) using a photoionization detector (PID). Upon opening of valve 1 (top panel), valve 2 (middle panel) or both valves (bottom panel), three odor pulses with a duration of 2 s each were emitted (grey shadows). (**b**) Representative SPME-GC-MS chromatograms of stimulation with benzaldehyde (red, top), ethyl acetate (blue-green, middle) and their binary mixture (orange, bottom). (**c**) PID responses to 2 s pulses of ethyl acetate and benzaldehyde at the different concentrations used in this study.

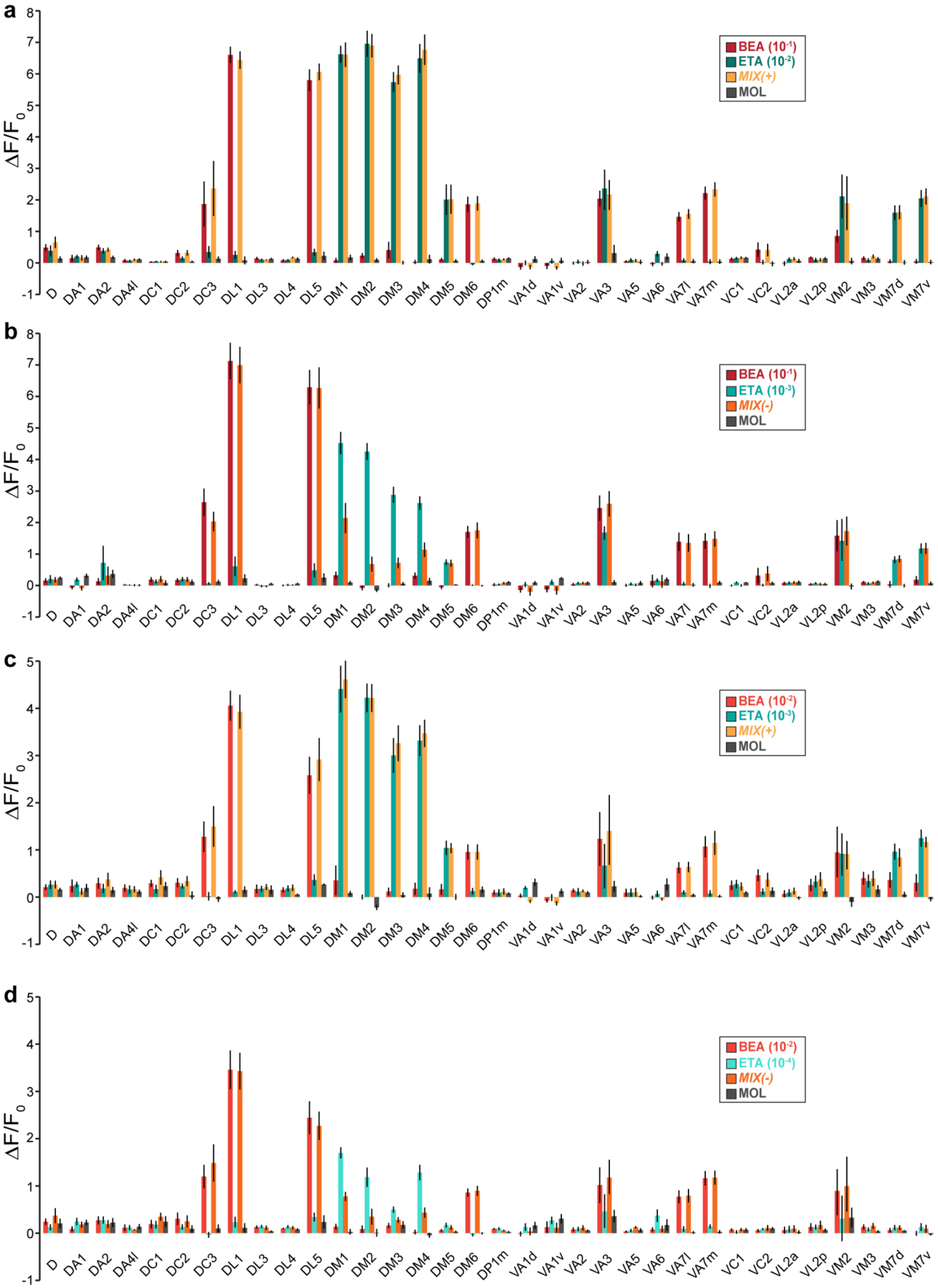
a
ΔF/F0
BEA (10^{-1})
ETA (10^{-2})
MIX(+)
MOL
D DA1 DA2 DA4l DC1 DC2 DC3 DL1 DL3 DL4 DL5 DM1 DM2 DM3 DM4 DM5 DM6 DP1m VA1d VA1v VA2 VA3 VA5 VA6 VA7l VA7m VC1 VC2 VL2a VL2p VM2 VM3 VM7d VM7v
b
ΔF/F0
BEA (10^{-1})
ETA (10^{-3})
MIX(-)
MOL
D DA1 DA2 DA4l DC1 DC2 DC3 DL1 DL3 DL4 DL5 DM1 DM2 DM3 DM4 DM5 DM6 DP1m VA1d VA1v VA2 VA3 VA5 VA6 VA7l VA7m VC1 VC2 VL2a VL2p VM2 VM3 VM7d VM7v
c
ΔF/F0
BEA (10^{-2})
ETA (10^{-3})
MIX(+)
MOL
D DA1 DA2 DA4l DC1 DC2 DC3 DL1 DL3 DL4 DL5 DM1 DM2 DM3 DM4 DM5 DM6 DP1m VA1d VA1v VA2 VA3 VA5 VA6 VA7l VA7m VC1 VC2 VL2a VL2p VM2 VM3 VM7d VM7v
d
ΔF/F0
BEA (10^{-2})
ETA (10^{-4})
MIX(-)
MOL
D DA1 DA2 DA4l DC1 DC2 DC3 DL1 DL3 DL4 DL5 DM1 DM2 DM3 DM4 DM5 DM6 DP1m VA1d VA1v VA2 VA3 VA5 VA6 VA7l VA7m VC1 VC2 VL2a VL2p VM2 VM3 VM7d VM7v

Supplementary Figure 2

Two-photon calcium imaging of PNs to binary mixtures of ethyl acetate and benzaldehyde.
(**a-d**) Mean PN activity of 34 identified glomeruli from 5-6 focal planes of flies expressing *UAS-GCaMP6s* under control of *GH146-Gal4* obtained at the 2-photon microscope upon stimulation with different combinations of single odors and their binary mixtures. Data represent mean ± SEM (n=6-11). BEA, benzaldehyde; ETA, ethyl acetate; MIX(+), attractive mixture; MIX(-), mixture with reduced attraction; MOL, mineral oil.

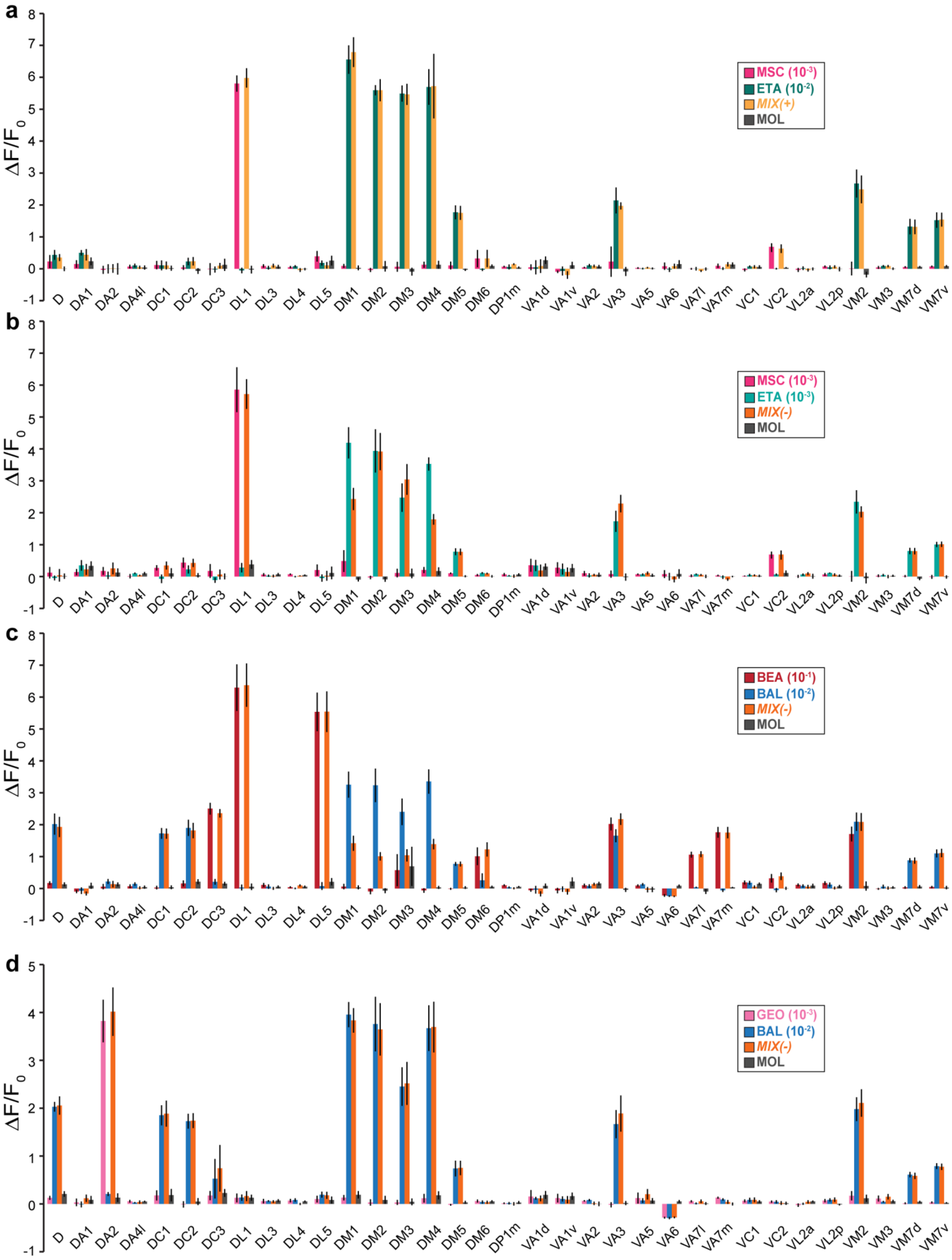
a
ΔF/F0
MSC (10-3)
ETA (10-2)
MIX(+)
MOL
b
ΔF/F0
MSC (10-3)
ETA (10-3)
MIX(-)
MOL
c
ΔF/F0
BEA (10-1)
BAL (10-2)
MIX(-)
MOL
d
ΔF/F0
GEO (10-3)
BAL (10-2)
MIX(-)
MOL
D DA1 DA2 DA4l DC1 DC2 DC3 DL1 DL3 DL4 DL5 DM1 DM2 DM3 DM4 DM5 DM6 DP1m VA1d VA1v VA2 VA3 VA5 VA6 VA7l VA7m VC1 VC2 VL2a VL2p VM2 VM3 VM7d VM7v

Supplementary Figure 3

Two-photon calcium imaging of PNs to binary mixtures of odors with opposing valences.

(**a-d**) Mean PN activity of 34 identified glomeruli from 5-6 focal planes of flies expressing *UAS-GCaMP6s* under control of *GH146-Gal4* obtained at the 2-photon microscope upon stimulation with different combinations of single odors and their binary mixtures. Data represent mean ± SEM (n=6-11). BEA, benzaldehyde; ETA, ethyl acetate; MSC, methyl salicylate; BAL, balsamic vinegar; GEO, geosmin; MIX(+), attractive mixture; MIX(-), mixture with reduced attraction; MOL, mineral oil.

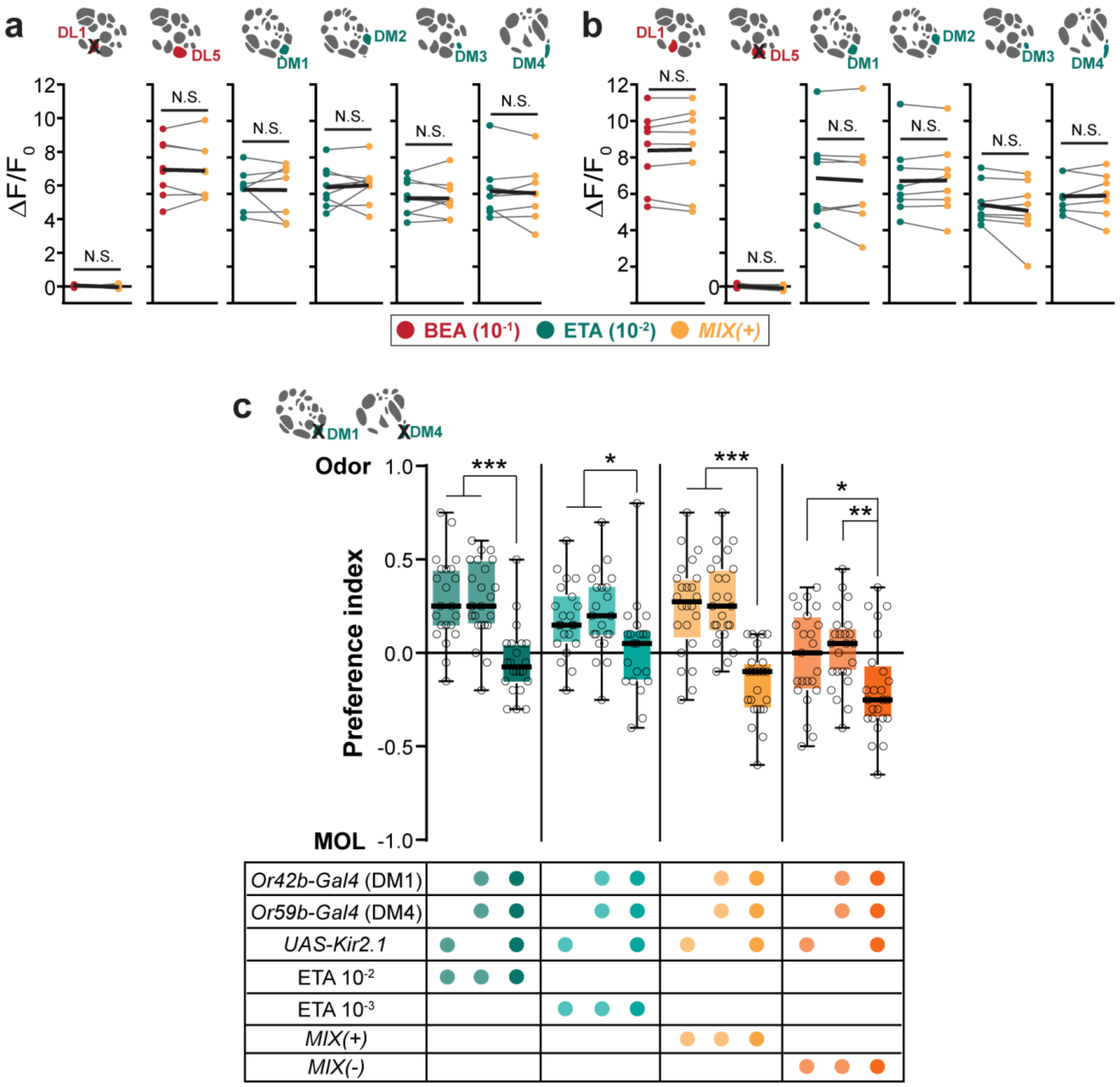

Supplementary Figure 4

Silencing the input to *repellent-responsive* and *attractant-responsive* glomeruli.

(**a**,**b**) Mean PN activity obtained with 2-photon imaging of the repellent- and attractant-responsive glomeruli during stimulation with ethyl acetate (10^{-2}, blue-green), benzaldehyde (10^{-1}, red) and MIX(+) (yellow) in Or10a$^{-/-}$ (a) and Or7a$^{-/-}$ (b) mutant flies. Individual flies are given by individual dots and lines; mean is indicated by black thick line (n=8, paired t-test). Pairwise comparisons of mixture responses to the response with the strongest single component (i.e. with either ethyl acetate or benzaldehyde) are shown for each animal. (**c**) Box plots showing behavioral attraction indices in the T-maze assay of flies with silenced or normal input to the *attractant-responsive* glomeruli DM1 and DM4 to the odors ethyl acetate (10^{-2} / 10^{-3}), MIX(+) or MIX(-) against the solvent control (MOL). Silencing of glomeruli DM1 and DM4 was achieved by overexpression of the potassium channel *Kir2.1* in Or42b- and Or59b-expressing ORNs. Treatment and genotypes are indicated by the table below the graph. Black line in the box represents median (n=21-24, one-way ANOVA with posthoc Dunnett's multiple comparisons test, *p<0.05,**p<0.01,***p<0.001). MIX(+) and MIX(-) induce an aversive response (i.e. significantly different from zero, student's t-test, ***p<0.001).

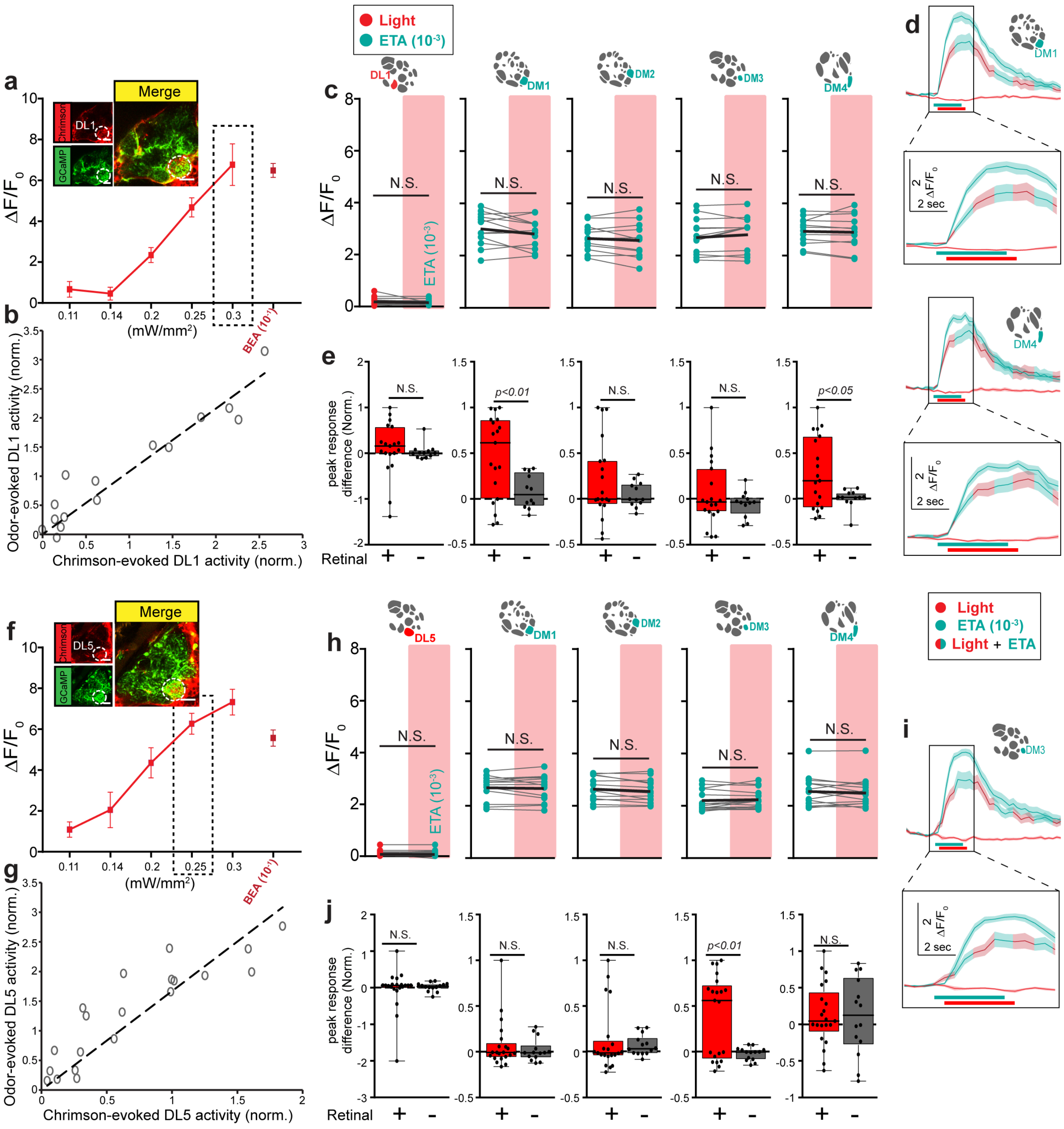

Supplementary Figure 5

Control experiments for optogenetic activation using CsChrimson.

(**a,f**) Odor- and light-evoked activity in PNs of flies expressing CsChrimson in ORNs of glomeruli DL1 or DL5, respectively. Light and odor responses are averaged across three trials for DL1 and four trails for DL5, error bars give SEM. Dotted box marks the light intensity equivalent to activity evoked by benzaldehyde (10^{-1}) and is used in all subsequent imaging experiments in both cases. Insets represent colocalization of CsChrimson (red) in ORNs of DL1 or DL5 and GCaMP (green) in PNs. (**b,g**) Calcium signals in glomerulus DL1 (b) or DL5 (g) evoked by light are nearly linearly correlated to

odor-evoked activity induced by benzaldehyde ($R^2 = 0.90$ for DL1 and $R^2 = 0.79$ for DL5). Signals are normalized to the maximum signal between trails (n=3 for DL1, n=4 for DL5). (**c,h**) Mean PN activity of the repellent- and attractant-responsive glomeruli during stimulation with either light (red dots), ethyl acetate (10^{-3}, bright blue-green dots) or both combined (additional red rectangles) in flies expressing CsChrimson in ORNs of glomerulus DL1 (c) or DL5 (h) and GCaMP3 in PNs. Flies have not been fed on all-trans retinal (n=12-14, paired t-test). (**d,i**) Close-up of averaged time traces of PN responses of glomeruli DM1 and DM4 (from Figure 5c) or glomerulus DM3 (from Figure 5f) to stimulation with light (red line), ethyl acetate (10^{-3}, bright blue-green line) or both (striped line). Shadows represent SEM. Blue-green bar represents odor stimulation, red bar light stimulation. The inhibition occurs at the onset of the light stimulus. (**e,j**) Box plots representing normalized peak response differences of calcium signals from attractant- and repellent-responsive glomeruli in in flies expressing CsChrimson in ORNs of glomerulus DL1 (e) or DL5 (j) either fed on cornmeal food supplemented with all-trans retinal (red) or without retinal (grey). Black lines represent median, dots show individual animals (n=12-21, students t-test).

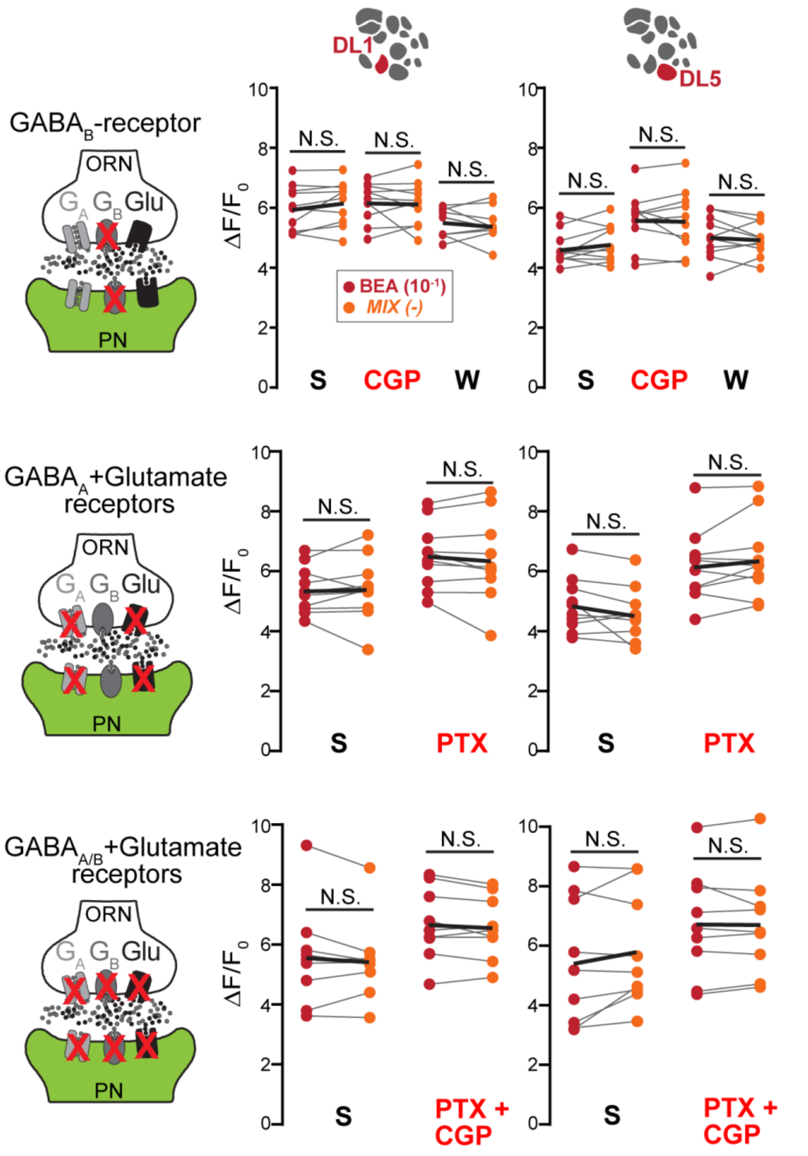

Supplementary Figure 6

***MIX(-)* responses of *repellent-responsive* glomeruli is not affected by application of GABA- and glutamate antagonists.**

Left, schematic drawing illustrating the experimental design: the $GABA_B$ antagonist CGP54626 (50 µM), $GABA_A$ and glutamate antagonist picrotoxin (100 µM) or a mixture of CGP54626 (50 µM) and picrotoxin (100 µM) is applied while calcium responses of PNs are monitored (green). *Right*, mean PN activity of the two repellent-responsive glomeruli (DL1 and DL5) during antagonist application (CGP, PTX or PTX+CGP) compared to saline (S) and wash-out (W) during stimulation with benzaldehyde (10^{-1}, BEA, red) and MIX(-) (orange). Individual flies are given by single dots and lines; mean is indicated by black thick line (n=10, paired t-test).

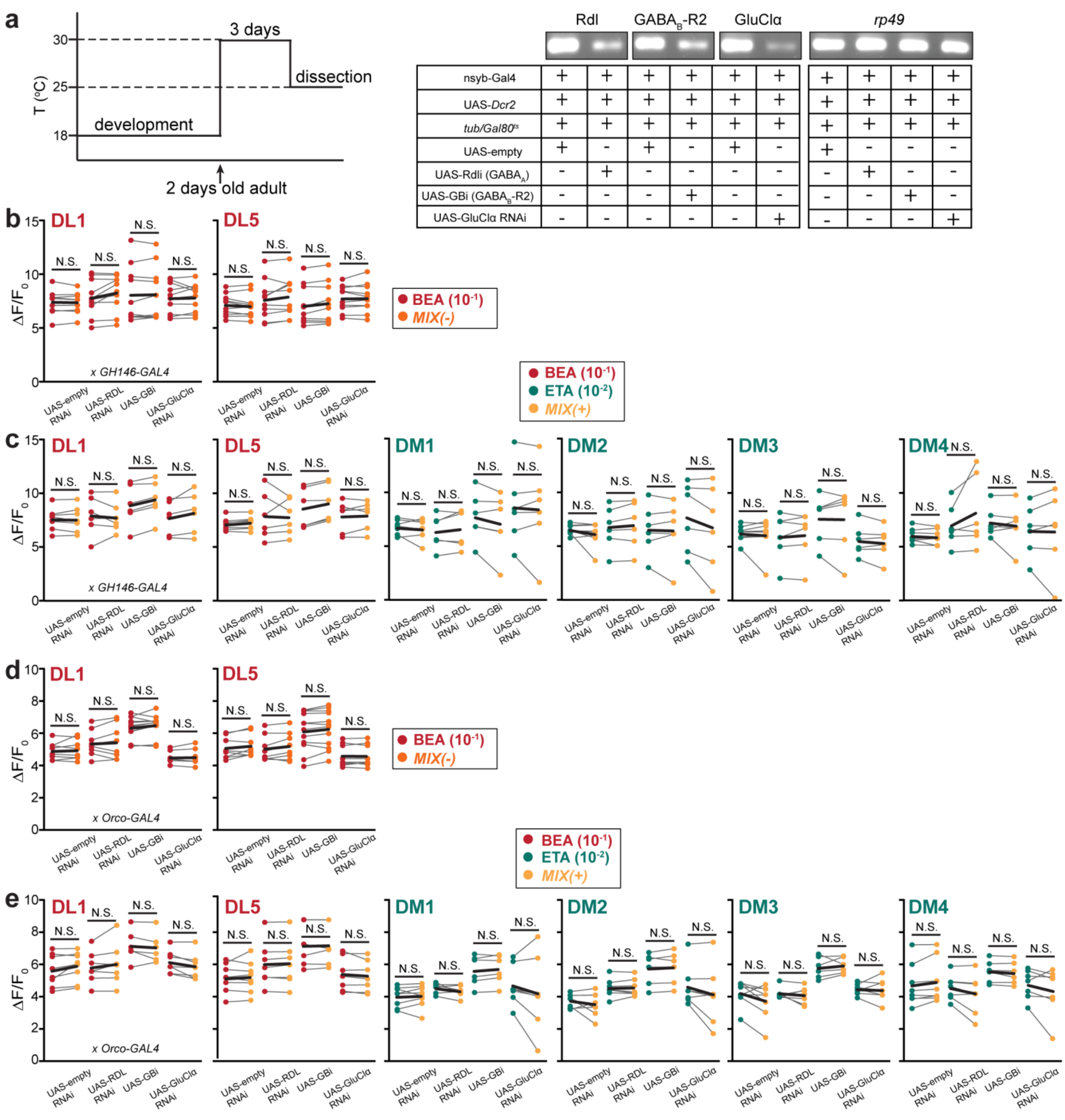

Supplementary Figure 7

Effect of RNAi lines to manipulate inhibitory pathways.

(**a**) *Left*, schematic of the experimental design. *Right*, products of RT-PCR reactions from 50-70 female heads bearing different RNAi lines (genotypes specified in Figure 7) with primers for GABA$_B$R2, GABA$_A$-Rdl subunit, Gluclα, and RP49 (gene for a ubiquitous ribosomal protein, used as an internal control). (**b**) Mean PN activity of the two repellent-responsive glomeruli (DL1 and DL5) during stimulation with benzaldehyde (10^{-1}, BEA, red) and MIX(-) (orange) in flies expressing different RNAi lines against GABA or glutamate receptors in PNs. Individual flies are depicted by single dots and lines; mean is indicated by black thick line (n=10, paired t-test). (**c**) Mean PN activity of the

attractant- and repellent-responsive glomeruli during stimulation with ethyl acetate (10^{-2}, ETA, blue-green), benzaldehyde (10^{-1}, BEA, red) and MIX(+) (yellow) in flies expressing different RNAi lines against GABA or glutamate receptors in PNs. Individual flies are given by single dots and lines; mean is indicated by black thick line (n=6, paired t-test). (**d,e**) Analogous to b,c, but RNAi lines were expressed in ORNs (n=6-12, paired t-test).

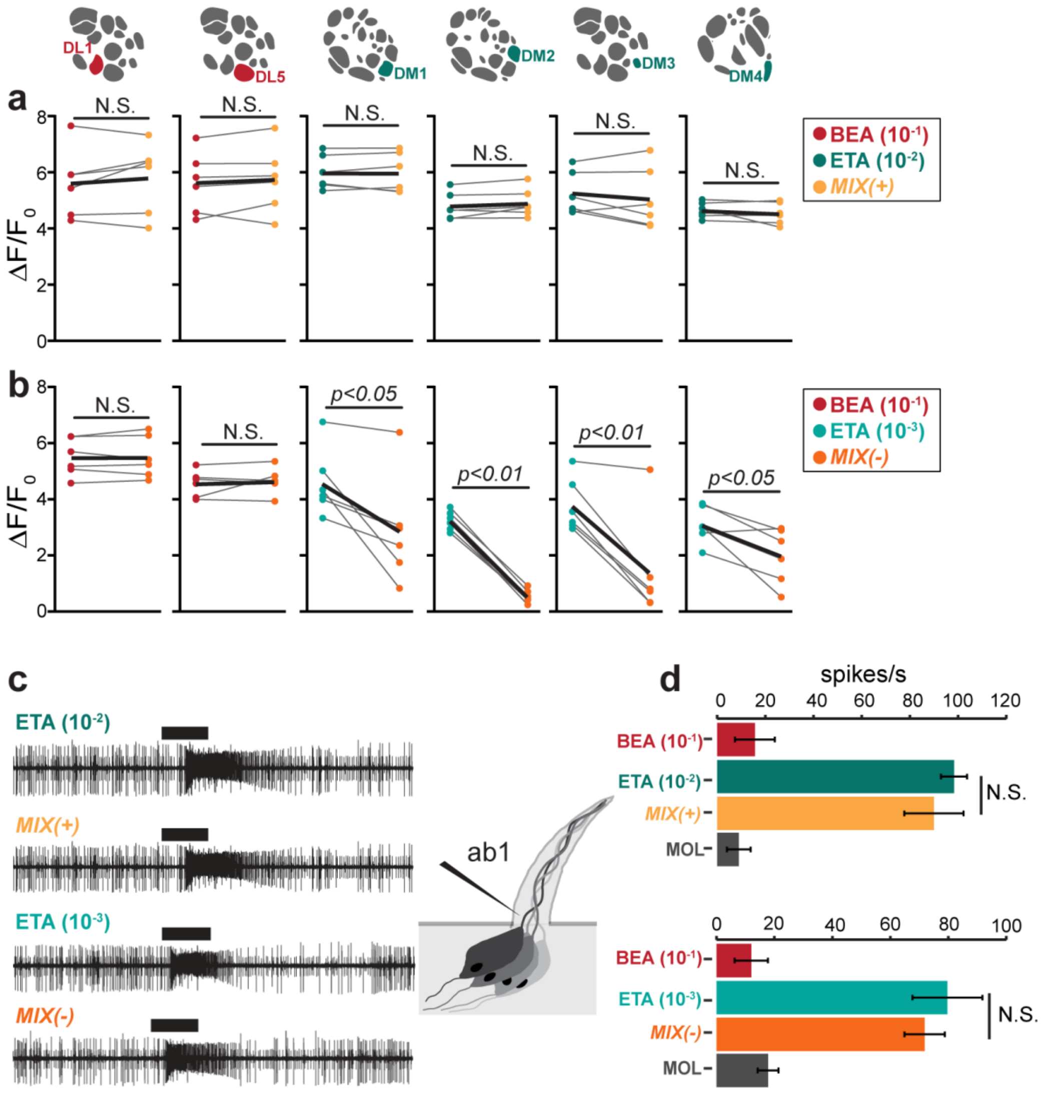

Supplementary Figure 8

MIX(-)-induced inhibition can be observed in ORNs but does not occur at the sensillum level.
(**a,b**) Mean PN activity of the attractant- and repellent-responsive glomeruli in flies bearing *UAS-GCaMP6f* under control of *Orco-Gal4* upon stimulation with two concentrations of ethyl acetate ($10^{-2}/10^{-3}$), benzaldehyde (10^{-1}), and both mixtures, MIX (+) and MIX(-). Individual flies are given by single dots and lines; mean is indicated by black thick line (n=6, paired t-test). (**c**) Representative SSR traces from an ab1 sensillum (which houses 4 ORNs) stimulated with two concentrations of ethyl acetate ($10^{-2}/10^{-3}$), MIX(+) and MIX(-). The duration of stimulus delivery (1s) is indicated by a black bar. (**d**) Quantified SSR responses to benzaldehyde (10^{-1}), ethyl acetate (10^{-2}), and their binary mixture (MIX(+)) (top panel) and to benzaldehyde (10^{-1}), ethyl acetate (10^{-3}), and their binary mixture (MIX(-)) (bottom panel) from ab1A sensilla. Error bars represent SEM (n=4, paired t-test).

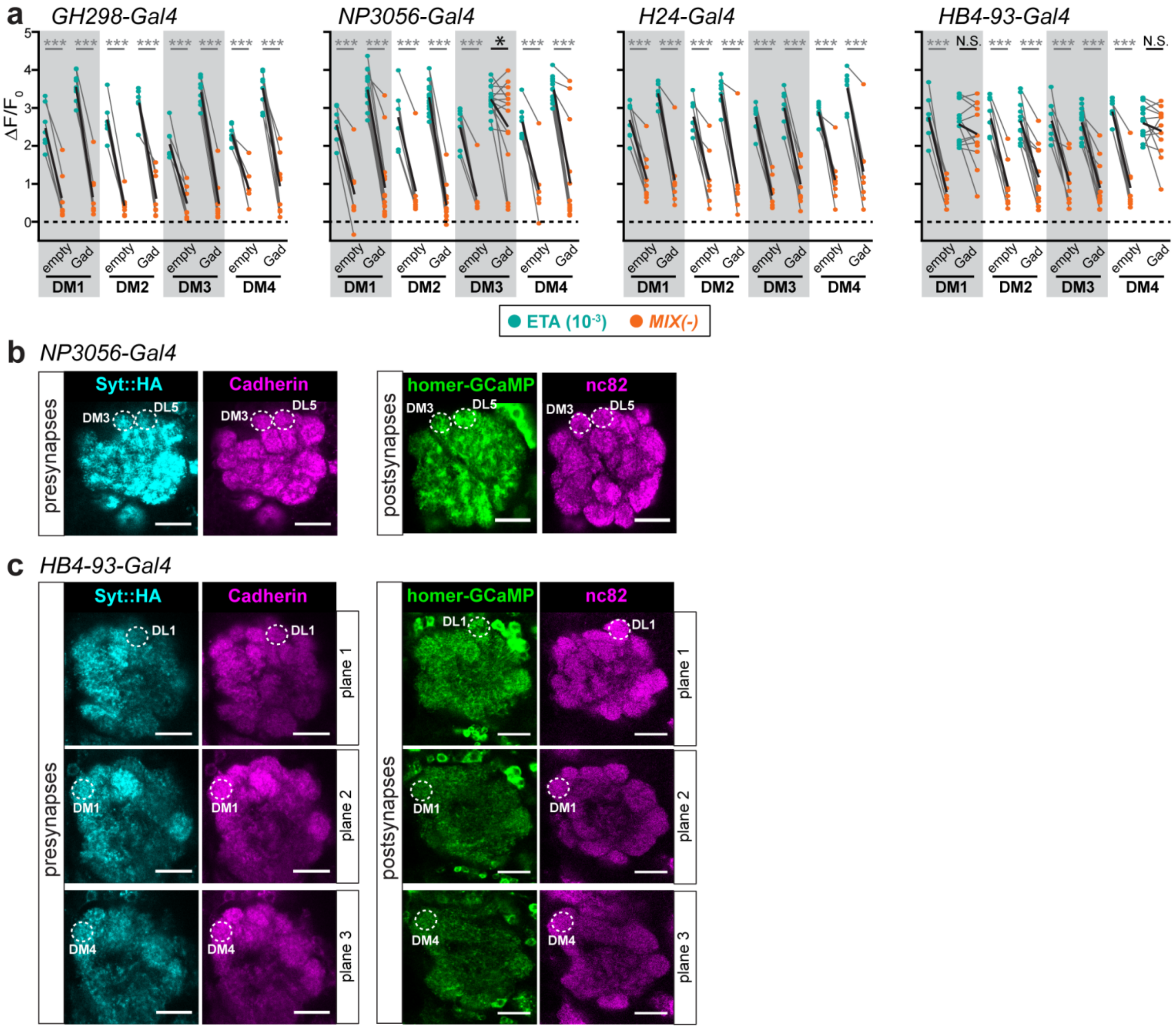

Supplementary Figure 9

Manipulation of GABAergic LN populations and investigation of their pre- and postsynaptic distribution.

(**a**) Mean PN activity of the four attractant-responsive glomeruli during stimulation with ethyl acetate (10^{-3}, bright blue-green) and MIX(-) (orange) in flies with intact (empty RNAi) or silenced (Gad RNAi) GABA production in four different LN lines (shown in Figure 8). Individual flies are given by individual dots and lines; mean is indicated by black thick line (n=7-15, paired t-test, *$p<0.05$, ***$p<0.001$).
(**b,c**) Immunostaining of pre- and postsynaptic densities in selected glomeruli (dotted circles) within the AL of two different Gal4 lines that label subpopulations of LNs. *Left*, immunostaining against the presynaptic protein syt::HA, which was expressed in LNs of *NP3056-Gal4* (b) or *HB4-93-Gal4* (c). *Right*, immunostaining against the postsynaptic marker homer combined with GCaMP3, which was expressed in LNs of *NP3056-Gal4* (b) or *HB4-93-Gal4* (c). Cadherin and nc82 immunostaining was used to facilitate glomerular identification. Scale bar 20µm.
