# Supplementary material for: Odor mixtures of opposing valence unveil inter-glomerular crosstalk in the Drosophila antennal lobe
Source: Nat Commun. 2019 Mar 13;10:1201. doi: 10.1038/s41467-019-09069-1 (PMC6416470; doi:10.1038/s41467-019-09069-1)
Supplement: Supplementary file 3 — Reporting Summary [file 41467_2019_9069_MOESM3_ESM.pdf]

nature research

Corresponding author(s): Dr. Silke Sachse

# Reporting Summary

Nature Research wishes to improve the reproducibility of the work that we publish. This form provides structure for consistency and transparency in reporting. For further information on Nature Research policies, see Authors & Referees and the Editorial Policy Checklist.

## Statistical parameters

When statistical analyses are reported, confirm that the following items are present in the relevant location (e.g. figure legend, table legend, main text, or Methods section).

| n/a | Confirmed | |
|---|---|---|
| ☐ | ☒ | The exact sample size ($n$) for each experimental group/condition, given as a discrete number and unit of measurement |
| ☐ | ☒ | An indication of whether measurements were taken from distinct samples or whether the same sample was measured repeatedly |
| ☐ | ☒ | The statistical test(s) used AND whether they are one- or two-sided<br>*Only common tests should be described solely by name; describe more complex techniques in the Methods section.* |
| ☐ | ☒ | A description of all covariates tested |
| ☐ | ☒ | A description of any assumptions or corrections, such as tests of normality and adjustment for multiple comparisons |
| ☐ | ☒ | A full description of the statistics including central tendency (e.g. means) or other basic estimates (e.g. regression coefficient) AND variation (e.g. standard deviation) or associated estimates of uncertainty (e.g. confidence intervals) |
| ☐ | ☒ | For null hypothesis testing, the test statistic (e.g. $F$, $t$, $r$) with confidence intervals, effect sizes, degrees of freedom and $P$ value noted<br>*Give P values as exact values whenever suitable.* |
| ☒ | ☐ | For Bayesian analysis, information on the choice of priors and Markov chain Monte Carlo settings |
| ☒ | ☐ | For hierarchical and complex designs, identification of the appropriate level for tests and full reporting of outcomes |
| ☒ | ☐ | Estimates of effect sizes (e.g. Cohen's $d$, Pearson's $r$), indicating how they were calculated |
| ☐ | ☒ | Clearly defined error bars<br>*State explicitly what error bars represent (e.g. SD, SE, CI)* |

*Our web collection on statistics for biologists may be useful.*

## Software and code

Policy information about availability of computer code

| | |
|---|---|
| Data collection | no software was used |
| Data analysis | Imaging data were analyzed with custom written IDL software (ITT Visual Information Solutions) provided by Mathias Ditzen (Stökl et al., 2010; Strutz et al., 2012). Immunostaining scans were analyzed using Image J. FlyWalk data were analyzed using custom code written in R. All graphs and statistcal analysis were performed using custom code written in R, GraphPad Prism 7, Metaboanalyst https://www.metaboanalyst.ca/MetaboAnalyst/faces/home.xhtml or Excel. |

For manuscripts utilizing custom algorithms or software that are central to the research but not yet described in published literature, software must be made available to editors/reviewers upon request. We strongly encourage code deposition in a community repository (e.g. GitHub). See the Nature Research guidelines for submitting code & software for further information.

## Data

Policy information about availability of data

All manuscripts must include a data availability statement. This statement should provide the following information, where applicable:

- Accession codes, unique identifiers, or web links for publicly available datasets
- A list of figures that have associated raw data
- A description of any restrictions on data availability

The datasets generated during and/or analysed during the current study are available from the corresponding author on reasonable request.

# Field-specific reporting

Please select the best fit for your research. If you are not sure, read the appropriate sections before making your selection.

☒ Life sciences ☐ Behavioural & social sciences ☐ Ecological, evolutionary & environmental sciences

For a reference copy of the document with all sections, see nature.com/authors/policies/ReportingSummary-flat.pdf

# Life sciences study design

All studies must disclose on these points even when the disclosure is negative.

| | |
|---|---|
| Sample size | We predetermined sample sizes not using any statistical methods but based on effect sizes, sample-by-sample variability observed in pilot experiments and literatures in the field. |
| Data exclusions | Recordings were terminated and we excluded the data when animals failed to show any responses to odor stimulation in the whole antennal lobe, or when the animal died before completing the whole experimental set. In case of artificial activation (Chrimson), data were excluded when animals (+ all-trans Retinal) showed no response in the corresponding glomerulus to the light stimulation. |
| Replication | The experimental findings were reliably reproduced as shown in the figures where we showed the entire distribution of data. Individual examples as well as population means are presented. We showed only example data for the results of immunostaining experiments because the high repeatability of the method (Gal4-UAS based fluorescent protein expression) has been widely recognized in the field. |
| Randomization | No randomization was used. |
| Blinding | Data collection and analysis were not performed blind to the conditions of the experiments. |

# Reporting for specific materials, systems and methods

### Materials & experimental systems

| n/a | Involved in the study | |
|---|---|---|
| ☒ | ☐ | Unique biological materials |
| ☐ | ☒ | Antibodies |
| ☒ | ☐ | Eukaryotic cell lines |
| ☒ | ☐ | Palaeontology |
| ☐ | ☒ | Animals and other organisms |
| ☒ | ☐ | Human research participants |

### Methods

| n/a | Involved in the study | |
|---|---|---|
| ☒ | ☐ | ChIP-seq |
| ☒ | ☐ | Flow cytometry |
| ☒ | ☐ | MRI-based neuroimaging |

## Antibodies

| | |
|---|---|
| Antibodies used | chicken anti-GFP (Life Technologies Cat# A10262, RRID AB_2534023), mouse anti-HA (Abcam Cat# AB18181, RRID AB_444303), rat anti-Cadherin (Developmental Studies Hybridoma Bank [DSHB] Cat# DCAD2, RRID:AB_528120), rabbit anti-GABA (Sigma Cat# A2052, RRID:AB_477652), mouse mAb anti-bruchpilot (nc82) (DSHB Cat# nc82, RRID: AB_2314865), Alexa Fluor 488 goat anti-chicken IgY (Life Technologies Cat# A-11039, RRID AB_2534096), Alexa Fluor 568 goat anti-rabbit IgG (Life Technologies Cat# A-11011, RRID AB_143157), Alexa Fluor 633 goat anti-mouse (Life Technologies Cat# A-21052, RRID AB_2535719). All antibodies are widely used in the field. |
| Validation | The validation data are reported by the companies that provide the antibodies. |

## Animals and other organisms

Policy information about studies involving animals; ARRIVE guidelines recommended for reporting animal research

| | |
|---|---|
| Laboratory animals | Drosophila melanogaster, females, 4-6 days after eclosion (unless otherwise mentioned) (different genotypes - see Materials and Methods) |
| Wild animals | N/A |
| Field-collected samples | N/A |
